# A multi-tissue longitudinal proteomics study to evaluate the suitability of post-mortem samples for pathophysiological research

**DOI:** 10.1038/s42003-025-07515-z

**Published:** 2025-01-17

**Authors:** Christian M. Beusch, Ken Braesch-Andersen, Ulrika Felldin, Pierre Sabatier, Anna Widgren, Jonas Bergquist, Karl-Henrik Grinnemo, Sergey Rodin

**Affiliations:** 1 Cardio-Thoracic Translational Medicine (CTTM) Lab, Department of Surgical Sciences, Uppsala University, Uppsala, Sweden; 2 Department of Pathology and Laboratory Medicine, Emory University, Atlanta, GA USA; 3 Novo Nordisk Foundation Center for Protein Research, University of Copenhagen, Copenhagen, Denmark; 4 Department of Chemistry – BMC, Analytical Chemistry and Neurochemistry, Uppsala University, Uppsala, Sweden; 5 Department of Cardio-Thoracic Surgery and Anesthesiology, Uppsala University Hospital, Uppsala, Sweden

**Keywords:** Proteomics, Proteomic analysis

## Abstract

Recent developments in mass spectrometry-based proteomics have established it as a robust tool for system-wide analyses essential for pathophysiological research. While post-mortem samples are a critical source for these studies, our understanding of how body decomposition influences the proteome remains limited. Here, we have revisited published data and conducted a clinically relevant time-course experiment in mice, revealing organ-specific proteome regulation after death, with only a fraction of these changes linked to protein autolysis. The liver and spleen exhibit significant proteomic alterations within hours post-mortem, whereas the heart displays only modest changes. Additionally, subcellular compartmentalization leads to an unexpected surge in proteome alterations at the earliest post-mortem interval (PMI). Additionally, we have conducted a comprehensive analysis of semi-tryptic peptides, revealing distinct consensus motifs for different organs, indicating organ-specific post-mortem protease activity. In conclusion, our findings emphasize the critical importance of considering PMI effects when designing proteomics studies, as these effects may significantly overshadow the impacts of diseases. Preferably, the samples should be taken in the operation room, especially for studies including subcellular compartmentalization or trans-organ comparison. In single-organ studies, the planning should involve careful control of PMI.

## Abstract (web summary)

Longitudinal proteomics study identifies variability in proteome alterations in different tissues, reveals distinct consensus semitryptic motives in different organs and provides criteria for designing proteomic studies involving post-mortem samples.

## Introduction

Comprehensive analysis of biomolecules serves as a robust foundation for scientific research in molecular biology and, particularly, for unraveling the pathophysiology of diseases. Until recently, transcriptomics has been the primary method for system-wide research due to the unparalleled depth of the datasets^1–4^. However, recent advances in liquid chromatography coupled with tandem mass spectrometry (LC-MS/MS) have revolutionized the field by dramatically increasing analytical depth and sample throughput. Contemporary mass-spectrometry systems facilitate the analysis of dozens of samples per day, with high quantification accuracy and analytical depth, reaching more than ten thousand proteins per sample for “bulk” analyses and many thousands of proteins per single cell^5–9^. Moreover, proteomics offers the capability to analyze a diverse array of post-translational modifications, protein structural parameters, and subcellular localization, providing orthogonal information to the expression/abundance measurements^10–15^. In addition, the rapid adoption of data-independent acquisition (DIA) methods provides numerous advantages over the traditional data-dependent acquisition (DDA) mode that are especially useful for high-throughput analyses of very heterogeneous samples^16^. The advantages include the ability to detect low-abundant peptides, increased specificity, and higher reproducibility across sample cohorts. Given that proteins, rather than RNAs, drive biochemical processes and protein abundances do not always correlate with the corresponding mRNAs^17,18^, LC-MS/MS datasets will provide complementary information to often already existing transcriptomic datasets on biochemical and cellular processes in both normal development and disease^19–21^.

Biopsy samples derived from both living patients, for instance during surgical operations, and postmortem cadavers, encompassing tissues, cells, and bodily fluids, serve as rich repositories of molecular information^22–25^. These samples empower the exploration of the intricate molecular landscapes associated with pathological conditions pinpointing disease markers, biomolecules, and signaling pathways, thereby creating opportunities for the development of targeted therapeutic approaches. The rapid development of transcriptomics over the last two decades has yielded comprehensive quality criteria for the eligibility of the samples for quantitation^26,27^. The nascent area of deep and high-throughput LC-MS/MS-based proteomics majorly lacks the criteria that may reduce its predictive power. For instance, recently we have compared extracellular matrix samples from the aortic valve resected during the aortic valve replacement operation from patients with aortic valve degeneration and the main objection from the reviewers was the absence of healthy control samples^28^. The only feasible route to obtain healthy samples of the aortic valve from living people is via resection from the recipient’s heart during heart transplantation at the time of surgery. However, collecting a sufficient number of samples would take a significant amount of time. More importantly, since the hearts need to be transplanted, the tissues may be affected by various pathological conditions. Conversely, the validity of the quantitative comparison between easy-to-collect post-mortem control samples and experimental samples obtained from living individuals is questionable.

While biopsies or samples from surgically resected specimens provide a dynamic snapshot of real-time molecular alterations and allow for the investigation of intervention effects, they often come with limitations. Indeed, this comprises restricted tissue types and limited quantities, posing constraints for scientific studies. Alternatively, postmortem cadaveric sampling often overcomes these limitations regarding the number of specimens and the amount of tissue. Consonantly, these samples are commonly used when larger studies need to be conducted, particularly in the case of neurodegenerative or cardiovascular research^29–33^. However, when working with post-mortem samples additional bias and confounding effects should be considered^34^, including the cause of death but also the time until tissue samples are extracted, the latter is often referred to as the post-mortem interval (PMI).

Several studies have described biochemical changes in post-mortem samples using unbiased proteomic methods. Some authors have investigated the possibility of determining the PMI based on proteomics or metabolism signatures mostly by animal models or in human bones primarily for forensics^35–38^. Others have been more interested in identifying criteria of suitability for characterizing protein expression patterns or identifying differences in their samples of interest based on different PMIs^39,40^. Among those studies, Kocsmár et al. investigated the PMI effect on different organs in human cadaver samples^41^. Although their research revealed notable variations in protein degradation kinetics in various organs, the use of human cadavers introduced potential biases related to subject genotype, age, sex, and overall health status. Importantly, tracking and controlling the PMI of human samples proves challenging.

However, there is currently no longitudinal study employing an isogenic model to investigate protein alterations in post-mortem samples across multiple organs allowing for an unbiased analysis of the PMI effect on the proteome. Additionally, no assessment of the subcellular compartmentalization of post-mortem samples has been conducted. In this study, we address these limitations by employing an isogenic animal model with a real-life relevant death time course and analyzing it by contemporary DIA LC-MS/MS.

We not only demonstrate significant variations in the kinetics of protein alterations among various tissues but also highlight the potential bias introduced by subcellular compartmentalization in quantifying proteins in early post-mortem samples. Additionally, our findings indicate that protein degradation, assessed through semi-tryptic peptide and gene ontology enrichment analyses, does not explain all observed protein alteration changes, arguing for controlled protein regulation post-mortem in a time-dependent fashion. Overall, the results of our work may be useful for designing studies involved in understanding of disease mechanisms, biomarker discovery, and the development of targeted therapeutic interventions.

## Results

### Assortment of PMI compared to biological differences

**Fig. 1:**
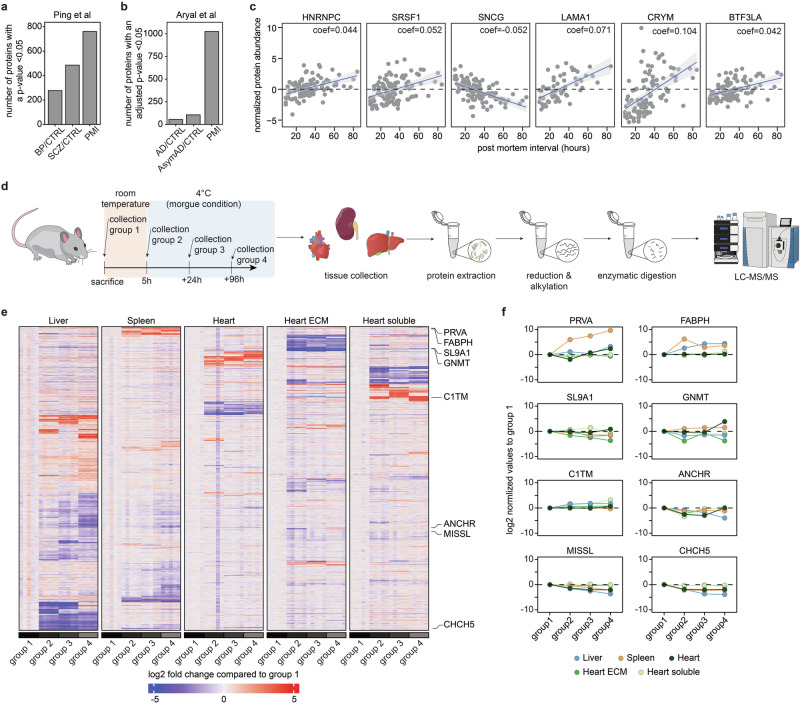
Multi-tissue longitudinal proteomics approach reveals drastic post-mortem proteome alterations. Assessment of the post-mortem interval (PMI) on protein alteration from Ping et al. (**a**) and Aryal et al. (**b**). **c** Selection of proteins exhibiting distinct protein level alteration post-mortem from Aryal et al. **d** Experimental design and workflow for proteomics measurement of post-mortem tissues in isogenic mice. **e** Heatmap of proteins with significant post-mortem alterations across the different tissues and subcellular fractions (*p* < 0.05 from one-sided ANOVA, absolute log2FC > 1). **f** Selection of protein with tissue-specific post-mortem protein alterations in mice. *N* = 5 for all mouse proteomics samples.

Before conducting experiments, we aimed to evaluate the influence of the PMI on protein abundance in comparison to the primary biological difference in previous studies. Utilizing publicly available proteomics data shared by the authors upon publication, we identified two datasets of human cadaver specimens that included PMI in the subject-specific metadata. One study by Ping et al. focused on understanding proteome changes in Alzheimer’s disease^42^. Employing a linear mixed effect model enables to concurrently evaluate the biological effect while adjusting for potential confounding factors. The biological comparison yielded 510 and 291 significantly differentially abundant proteins, respectively, while the PMI affected 799 proteins (Fig. 1a and Supplementary Data 1). In another study, human brain samples were examined to assess synaptic dysfunction in mental illnesses^43^. Here, the discrepancies were more pronounced, with 1026 proteins exhibiting a Benjamini-Hochberg adjusted *p* value < 0.05, compared to 106 and 55 in the biological groups being compared (Fig. 1b and Supplementary Data 2). Notably, proteins with the most significant differential abundance displayed distinct functions and subcellular localizations (Fig. 1c). For example, HNRNPC, SRSF1, and BTF3 are primarily localized in the nucleus and are involved in pre-mRNA processing, splicing factor activity, and transcriptional regulation, respectively. Conversely, LAMA1 is an extracellular protein and a key constituent of the basement membrane, or CRYM and SNCG are localized throughout the entire cell^44^.

### Design of a clinical realistic autopsy model

Since the reanalysis of the public datasets revealed significant potential for post-mortem protein alterations to confound the biological readouts, we conducted an analysis of a clinically relevant animal model, wherein various organs of major biomedical interest are harvested at precisely defined time intervals mirroring real-life scenarios in a hospital setting. Organs (hearts, livers, and kidneys) from the first group of mice are directly harvested upon sacrificing the animals, representing fresh samples that are taken in the operation theater (Fig. 1d). The remaining euthanized animals were kept at room temperature for 5 h, simulating the time elapsed between death and the transfer to a morgue, after which time the organs from the second group were collected. This time course also simulates the one from the Rapid Autopsy Program (RAP)^45,46^. The organs from the third group were extracted after 5 h at room temperature and 24 h at 4 °C, while the fourth group was collected after 5 h at room temperature and 96 h at 4 °C, representing cadavers with extensive time in the morgue before sample collection. However, it is not uncommon for samples to be used for research after 4 days in the morgue. After all the samples were collected, they were prepared for mass spectrometry analysis, and data acquisition was performed by DIA. In the case of the heart, after homogenization, a part of each sample was used for subcellular compartmentalization^28^. For simplicity, we use term heart samples for samples prepared from the whole heart; and term soluble heart samples and extracellular matrix (ECM) heart samples for samples prepared from cytosolic heart proteins and ECM proteins, respectively. Initial quality control measurements show close clustering of fresh samples by organ type and uniform protein quantification across the different tissues (Supplementary Fig. 1a). When comparing the analytical depth achieved by us with a pathophysiology study conducted on the same instrument using DDA, a notable enhancement in both protein and peptide levels was observed^41^. As demonstrated before, heart samples result in fewer protein identifications compared to the liver or spleen^47^ (Supplementary Fig. 1b, c). Interestingly, conducting an analysis of differentially regulated proteins unveiled significant changes post-mortem among the different tissues and subcellular fractions (Fig. 1e, Supplementary Fig. 1d, and Supplementary Data 3). While the majority of observed protein changes appear to be sample-specific, it is noteworthy that certain alterations are shared across multiple samples, indicating potential commonality in post-mortem effects across diverse biological contexts. PRVA, a calcium-binding protein that protects cells from reactive oxygen species, shows rapid protein abundance increase even 5 h post-mortem (group 2) in the spleen, while in the liver and heart, these changes are only significant in the late-stage cadaver samples^48^ (Fig. 1f). A comparable pattern is evident for FABP3, known for its inhibitory effect on proliferation and promotion of apoptosis^49,50^. It exhibits a rapid increase in protein abundance in the liver and spleen, while no discernible change is observed in samples originating from the heart. Notably, FABP3 has been identified as a potential biomarker for neurodegenerative diseases and is suggested to play a role in cardiac development^51,52^. The opposite behavior is observed for SLC9A1, a protein involved in the sensing of amino acid availability and subsequent signaling to mTORC1, however this time the heart ECM fraction reacts the fastest followed by the liver and spleen. Intriguingly, neither the soluble heart fraction nor the total heart exhibits any statistically significant changes. CHCH5, belonging to the CHD family of chromatin remodeling proteins and characterized as a pro-inflammatory polypeptide, shows a rapid abundance decrease in the spleen, liver, and heart^53^. These findings not only validate the robustness of our animal model but also underscore the critical importance of conducting a comprehensive tissue-specific analysis of post-mortem changes as a wide number of proteins with diverse functions are changing in abundance. The significance of this analysis extends to enhancing our understanding of these changes and highlighting potential biases that may impact subsequent studies.

### Organ-specific post-mortem difference

**Fig. 2:**
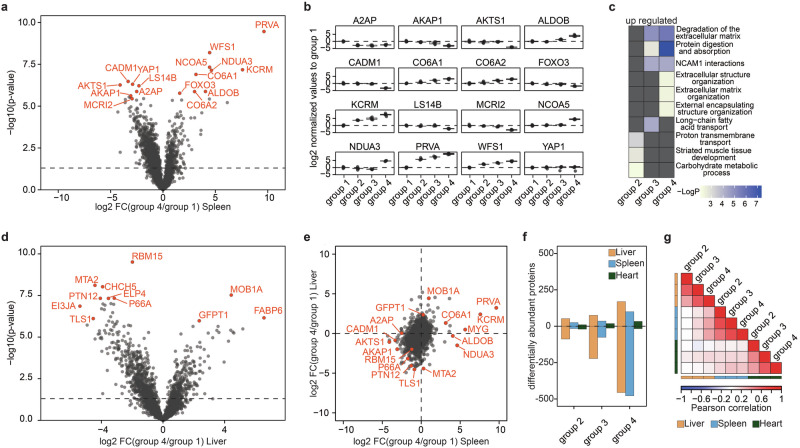
Organ-specific post-mortem difference. **a** Volcano plot highlights differentially abundant protein in the spleen between fresh samples (group 1) and samples incubated for 4 days. **b** Relative protein abundance changes of most significant changing protein in the spleen post-mortem. **c** Gene ontology analysis of upregulated proteins in the spleen in group 4 compared to group 1 (fresh). **d** Volcano plot highlights differentially abundant protein in the liver between fresh samples (group 1) and samples incubated for 4 days (group 4). **e** Scatter plot assessing the correlation of post-mortem changes between the spleen and liver. **f** Number of differentially regulated proteins post-mortem in the liver (yellow), spleen (blue), and heart (green). **g** Correlation analysis of protein alterations across the various tissues post-mortem. *P* values were calculated using a two-sided Student *t*-test using equal variance and a *p* < 0.05 was considered as significant. The horizontal line in the boxplots represents the median, 25th, and 75th percentiles and whiskers represent measurements to the 5th and 95th percentiles. *N* = 5 for all mouse proteomics samples.

Firstly, we aimed to examine the distinct proteome modifications occurring in each organ. Upon comparing protein alterations between freshly obtained tissues (group 1) and those stored for the longest duration (group 4) in the spleen, we observed consistent protein regulation (Fig. 2a and Supplementary Data 4). Notably, proteins exhibiting increased abundance over time include PRVA, KCRM, ADLDOB, and different collagens, while proteins such as AKTS1, FOXO3, and AKAOP1 demonstrated decreased abundance. KCRM plays a pivotal role in energy-demanding conditions by catalyzing the transfer of phosphate between ATP and various phosphogens like creatine phosphate^54^. ALDOB, also upregulated, suppresses AKT1 activity, whereas AKTS1, a key regulator of AKT1, exhibits lower expression levels in older samples^55^. FOXO3 regulates proteostasis and induces autophagy through AKT1^56^. Interestingly, muscle-specific deletion of FOXO members was found to protect against muscle loss due to their involvement in autophagy-lysosome and ubiquitin-proteasome systems^57^. Overall, these findings suggest a deficiency in nutrients and the requirement for alternative energy sources in the late samples. In addition, proteins like CO4A1, CO6A1, and CO6A2, all predominate localized as part of the extracellular matrix, become more abundant^44^. This specific change in the extracellular matrix could indicate a higher resistance of often heavily glycosylated extracellular matrix proteins to the increased enzymatic activity post-mortem. Interestingly, the temporal kinetics of the most significantly regulated proteins varied greatly (Fig. 2b). Proteins like PRVA or KCRM exhibited rapid and robust regulation, while those involved in AKT1 regulation and autophagy showed predominant changes in group 4 samples, possibly indicating different mechanisms to cope with resource scarcity. Gene Ontology enrichment analysis revealed clear pathway distinctions among the most significantly upregulated proteins, with early time points enriched in terms related to short-term energy maintenance and later time points associated with extracellular matrix degradation (Fig. 2c). Subsequently, we aimed to compare protein alterations across different organs. In the liver, notable abundance alterations were observed for MOB1A, FABP6, GFPT1, and MTA2, with TLS1 and EIF3J1 exhibiting increased or decreased abundance in long-term samples, respectively (Fig. 2d and Supplementary Fig. 2a). GFPT1, the rate-limiting enzyme of the hexosamine biosynthesis pathway, showed increased abundance in both liver and brain post-mortem autopsies^58^. Downregulated proteins included EIF3J1, a member of the EIF3 complex essential for de novo protein synthesis, and MTA2 and TTLS1, both implicated in apoptosis prevention through different mechanisms^59,60^. Overall, we observed a time-dependent increase in differentially regulated proteins across all organs, with the spleen and liver exhibiting drastic changes in group 4 samples compared to the heart, which showed a lesser magnitude of change (Fig. 2e). These organ-specific changes align with findings in human cadavers, validating our experimental design^41^. Comparison of proteome changes between fresh samples (group 1) and group 4 samples in the spleen and liver revealed minimal correlation (Fig. 2f), with only a few proteins showing increased abundance in older samples across both organs. Notably, the majority of changes were organ-specific, underscoring the unique proteomic alterations occurring over time (Supplementary Fig. 2b–d). Interestingly, it was previously reported that the relationship between PMI and RNA stability is also highly tissue-dependent^27,61^. Furthermore, while protein alterations correlated within different time points of the same organ, minimal to no correlation was observed across different organs, highlighting the organ-specific nature of the degradome (Fig. 2g). In summary, post-mortem proteome changes follow organ-specific temporal kinetics, precluding generalization. We also analyzed sarcomere proteins in the three organs and, as expected, the heart exhibited the highest abundance of them in comparison to the other two organs (Supplementary Fig. 2e).

### Subcellular compartmentalization of the degenerated heart tissue induces artifacts

**Fig. 3:**
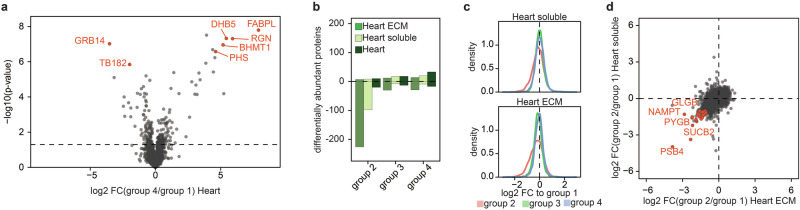
Post-mortem changes in the heart and its soluble and ECM subcellar compartment. **a** Volcano plot highlights differentially abundant protein in the heart between fresh samples (group 1) and samples incubated for 4 days. **b** Number of differentially regulated proteins post-mortem in the ECM, soluble fraction, and the total heart. **c** Distribution of protein alterations across various time points compared to fresh samples. **d** Scatter plot of protein changes in the subcellular fraction of the heart comparing group 2 and fresh samples. *P* values were calculated using a two-sided Student *t*-test using equal variance and a *p* < 0.05 was considered as significant. *N* = 5 for all mouse proteomics samples.

Previous studies have explored protein alterations following post-mortem changes, but a systematic investigation into the subcellular proteome has been lacking. In our analysis of full tissue samples, we observed significant protein deregulation during post-mortem decomposition in liver and spleen samples, while minimal changes were noted in the heart (Fig. 2e). Notably, the heart proteome remained remarkably stable even at the latest time point (group 4) (Fig. 3a and Supplementary Fig. 3a). Consequently, we aimed to examine protein degradation in the heart through subcellular compartmentalization using the same organs, following the same protocol as we have previously employed for the analysis of the extracellular proteome in AVD hearts^28^. Interestingly, the protein alterations in the soluble and extracellular matrix (ECM) fractions differed significantly from those in total heart samples. While the numbers of proteins with statistically significant differences in abundance in the fractions and the whole heart were similar in groups 3 and 4, an increase in proteins with lower abundance was observed in both heart fractions in group 2. However, this trend was corrected at latter time points, resulting in only a marginal overall decrease in protein abundance. This stands in contrast to samples from whole organs, where decreased protein abundance was primarily detected in later post-mortem samples (Supplementary Fig. 3b–e). These findings suggest a general dysregulation of protein localization immediately post-mortem, potentially as an extreme measure to compensate for changes in the cellular environment. Indeed, a comparison of protein alterations between the ECM and soluble heart fractions revealed a significant overlap in the most downregulated proteins (Fig. 3d).

### Post-mortem protein degeneration results in specific semi-tryptic peptides

**Fig. 4:**
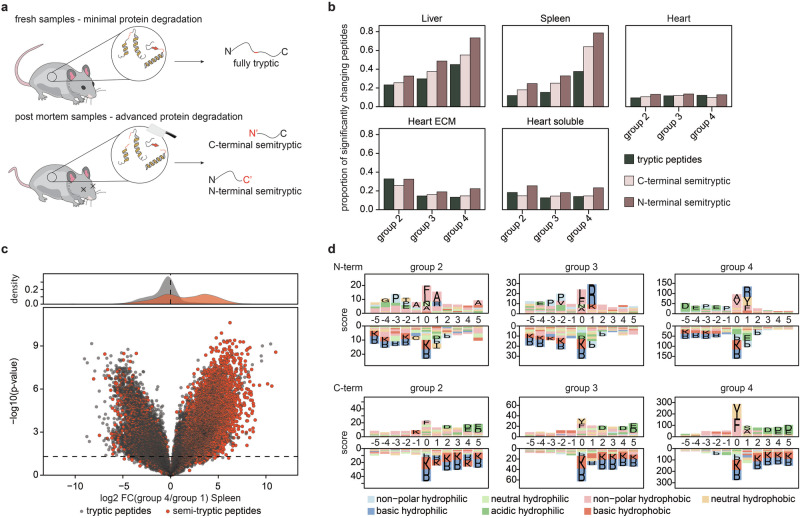
Analysis of the post-mortem degradome. **a** Concept of protein degradation post-mortem. **b** Number of significantly changing peptides post-mortem classified into their cleavage specificity. **c** Volcano plot highlights differentially abundant peptides in the spleen between fresh samples (group 1) and samples incubated for 4 days. **d** Semi-tryptic cleave motif enrichment analysis, reveals specific and time-dependent cleavage motifs. *P* values were calculated using a two-sided Student *t*-test using equal variance and a *p* < 0.05 was considered as significant. *N* = 5 for all mouse proteomics samples.

Proteome alterations in post-mortem samples, as compared to fresh samples, typically exhibit enrichment in protein downregulation, as observed by us and others in previous studies^36,41^. This phenomenon is commonly attributed to the activity of proteases in the tissues, leading to a time-dependent degradation of full-length proteins into smaller fragments (Fig. 4a). However, to date, there has not been a systematic assessment of these protease activities. This is primarily because most past studies relied on antibody-based readouts, such as Western Blots, or DDA proteomics methods. While the latter can in principle analyze semi-specific peptides, the precursor fragmentation logic of these methods often excludes the identification of them. In this study, we utilized a DIA method, ignoring precursor charge states, that enabled us to quantify more semi-tryptic peptides. Indeed, searching our data with semi-specific cleavage specificity revealed ~10–40% of all quantified peptides to be semi-specific, and this distribution was remarkably uniform across the different time points (Supplementary Fig. 4a–c). Additionally, most of these semi-tryptic peptides are already evident in the fresh cadaver samples, with a notable surge in their overall abundance specifically observed in the spleen. However, when evaluating the proportion of significantly regulated peptides relative to the total number of peptides, we identified a time-dependent increase of significant semi-specific peptides in spleen and liver samples already in group 2, 5 h post-mortem (Fig. 4b and Supplementary Data 5). Interestingly, N-terminal semi-tryptic peptides seem to increase slightly more than C-terminal ones. Further, as expected, semi-tryptic peptides show an increase in abundance over time, while fully tryptic ones decrease, validating the hypothesis that semi-tryptic peptides are getting generated at the cost of fully tryptic ones (Fig. 4c). Interestingly when assessing the cleavage specificity of these semi-tryptic peptides identified in the spleen samples, a clear motif can be determined. While N-terminal peptides cleave preferable non-polar hydrophobic (e.g., Alanine or Valine) after acidic or neutral hydrophilic amino acids (e.g., Proline, Aspartic acid or Glutamic acid), C-terminal peptides adhere an even more specific cleavage motif, involving tyrosine followed by an acidic hydrophilic residue (Fig. 4d). Identifying a single protease or a combination of multiple proteases is not trivial, as information for non-model proteases is scarce^62,63^. Importantly, these cleavage motifs change for other tissues (Supplementary Fig. 4e, f). For N-terminal semi-tryptic peptides, the liver shows a clear enrichment of motif consisting of a non-polar hydrophobic amino acid (mainly leucine or alanine) followed by an arginine, while the heart one is mainly driven by a central phenylalanine residue. Since we were unable to link the semi-tryptic cleavage to a specific protease using an existing database, we addressed this knowledge gap by examining the abundance of endogenous exopeptidases and endopeptidases in the three tissues. In line with the trend seen in semi-tryptic peptides, the highest protease abundance was observed in the spleen, followed by the other two organs. Notably, we identified proteases that were more abundantly expressed in certain tissues. This analysis could provide a foundation for exploring the cleavage patterns of these proteases and comparing them to the semi-tryptic peptides quantified in our experiment (Supplementary Fig. 5a, b). Overall, this represents the first systematic analysis of the PMI on peptide quantification with an emphasis on semi-tryptic peptides, not only providing a direct experimental explanation for the decrease in protein abundance post-mortem but also highlighting the involvement of tissue-specific proteases for this phenome.

## Discussion

Pathophysiology of diseases in humans and animals often significantly differs, which necessitates the analysis of human samples. Due to recent methodological and technological advances, LC-MS/MS-based proteomics is becoming a major system-wide approach for unraveling the pathophysiology of diseases, identification of therapeutic targets, and designing diagnostic tools. While the availability of samples from living human beings is scarce, samples from cadavers often provide more flexibility in the type and amount of obtained material. In addition, we speculate that the recent increase in sample throughput, analytical depth, and decrease in the price of LC-MS/MS-based proteomics will significantly increase the use of post-mortem biopsy samples for pathology. However, the analysis is impeded by death, which affects protein expression, protein degradation rate, and the activity of the endogenous digestion enzymes. To evaluate the range of the confounding effect, we reanalyzed two publicly available datasets and the effect of PMI could be comparable with that of the phenotype of interest in the studies (Fig. 1a, b), which was in concert with earlier reports. These results underline the drastic effect of the PMI on protein expression that can have a significant confounding influence on the analysis of the data. Consequently, it is imperative to formulate criteria for the use of post-mortem samples in LC-MS/MS-based proteomics that will minimize the confounding effect of PMI and increase the validity of the biological finding.

One method to address the effect of PMI is the established Rapid Autopsy Programs (RAP) for the collection of samples. However, this is a very resource- and time-consuming process and, in many cases, is not feasible. Another approach implies finding a range of PMIs in which the effects are relatively small and can be disregarded. Several earlier studies have investigated the effect of PMI using human tissues. However, these samples introduced potential biases concerning the subject genetic background, age, sex, and overall health status to evaluate the impact of postmortem interval (PMI) on protein expression. It is crucial to note that monitoring and managing the PMI of human samples present significant challenges. We designed a clinically relevant mouse model that eliminates the above-mentioned biases and accounts for standard treatment of cadavers including an initial stay at room temperature and a prolonged stay at the morgue. We collected fresh samples taken immediately after sacrificing the animals (group 1) and using LC-MS/MS compared them to samples taken after 5 h at room temperature (group 2) an additional 24 h (group 3) and 96 h (group 4) at +4 °C. Group 2 represented the earliest possible opportunity for taking samples from cadavers while groups 3 and 4 represented a standard range of timings for autopsies in a hospital. Crucially, in this study, we introduced a DIA-based proteomics approach, which is better suited for comprehensive system-wide analysis compared to traditional DDA methods. In addition, utilizing a DIA method enabled us to conduct a thorough examination of semi-tryptic peptides, enriching the overall analysis. To complement earlier studies on the effect of PMI on human cadavers, we used isogenic mice that allowed us to reduce variability related to genotype, age, and overall health status. The overall number of identified tryptic peptides and semi-tryptic peptides was stable for all PMIs (groups 2–4) in comparison with the fresh samples (group 1) for all organs and fractions except for the spleen (Supplementary Fig. 4a). For the spleen, the numbers were stable for all groups except for group 4, which exhibited an increase in the number of semi-tryptic peptides and a decrease in the number of tryptic peptides in comparison with those of all other groups. Since group 4 represents the longest PMI, this is an expected outcome. Also, the difference suggests significantly higher overall degradation of proteins in the spleen compared to the liver and the heart in which the numbers of tryptic and semi-tryptic peptides did not exhibit any significant difference at the same PMI (in group 4). Interestingly, the proportion of identified semi-tryptic peptide numbers to tryptic peptide numbers was significantly higher in the liver than in the spleen, but it stayed stable even at the latest tested PMI (group 4). We also compared the expression (abundance) of the detected proteins in the tissues at various PMIs (groups 2-4) in comparison with the fresh tissue (group 1) (Fig. 2e). The liver demonstrated a sharp increase in the number of differently expressed proteins starting from the earliest PMI exhibiting dozens of them already in group 2 (Fig. 2e). The spleen showed little change in group 2, dozens of proteins in group 3 and hundreds in group 4 (Fig. 2e). Although the liver and the spleen exhibited a similar number of differently expressed proteins in group 4 and an expected increase in the numbers with higher PMIs, a significant in the proteome is detected starting from group 2 and group 3 for the liver and the spleen, respectively. Unexpectedly, the heart did not demonstrate a significant change in the proteome and the number of differently expressed proteins was low and similar for all the PMIs. We also observed tissue-specific alterations of abundance for certain proteins such as PRVA, FABPH, and GNMT (Fig. 1f) that provided another evidence of tissue specificity in post-mortem proteome alterations. Indeed, a similar observation regarding the tissue-specific degradome was made by Ferreira et al., assessing the effect of the PMI on the quality of RNA transcripts, finding a large heterogeneity across a significant number of different human tissues and organs^27^.

The relative stability of the post-mortem heart proteome is curios and cannot be fully explained by the difference in the peptidase abundances (Supplementary Fig. 5a, b). We speculate that the accumulation of exceptionally high amounts of the lactic acid in cardiomyocytes under hypoxia caused by the death with subsequent release from the dying cells may inhibit the proteases via change in pH. Also, the heart is rich in the ECM and sarcomere proteins (Supplementary Fig. 2e) that may additionally impede proteolysis of the organ.

The compartmentalization is a fractionation method enabling the isolation of proteins from distinct cellular compartments, such as cytosolic, membrane, cytoskeleton, and extracellular fractions. It is frequently employed to investigate molecule translocations within the cell or for focused analyses of specific compartments. We isolated cytosolic (soluble) and ECM fractions of the heart^28^ and compared the abundance of the detected proteins at various PMIs (Fig. 3b). Surprisingly, both fractions exhibited a sharp increase in the number of proteins with reduced abundance in group 2 and low numbers of differently expressed proteins in groups 3 and 4.

To investigate the molecular underpinnings of the variation in protein abundance dynamics, we examined protein integrity by analyzing peptide cleavage specificity. Significantly, our analysis revealed a disparity in protease cleavage specificity between the liver and spleen samples during the late postmortem interval (PMI). Associating these cleavage motifs with a particular protease is not straightforward, despite recent research focusing on non-standard proteases^62,63^. It’s crucial to note that various proteases may contribute to the generation of semi-specific peptides, resulting in an overlap of distinct cleavage patterns. To draw a conclusion on which proteases are showing increased activity post-mortem will demand an additional comprehensive study. However, the difference in the cleavage specificity suggests that tissue-specific proteases are responsible for these changes. Importantly, since tissue-specific proteases are responsible for generating semi-tryptic peptides, each post-mortem tissue needs to be assessed from a specific view, and generalization of tissue behavior might result in bias.

We used the isogenic mouse model for the study and the analysis may yield even more heterogenous results in the diverse human populations. Our findings align with observations of organ-specific proteome alterations during post-mortem intervals in human cadaver studies^41^supporting the relevance of mice as a model organism for these investigations. The main conclusions of the study suggest limitations in the use of cadaver samples allowing the direct translation of the results to the human research. The findings that indicate relative stability of the mouse proteomes with increased PMIs should be confirmed with human samples before the translation.

In conclusion, our data suggest that while the individual might be dead, the tissues are not and may undergo significant tissue-specific proteome alterations even within several hours post-mortem. The difference in the consensus cleavage motifs in semi-tryptic peptides and tissue-specific dynamics of alterations of the same proteins suggest that tissue-specific fermentative systems rather than different susceptibility to autolysis are responsible for the degradation of the post-mortem tissues. Although, except for the spleen at the late PMI, we did not detect any significant increase in the number of semi-tryptic peptides there was a notable presence of proteins exhibiting changed abundance levels even during the early PMI in the spleen and liver, whereas such alterations were not observed in the heart. Therefore, in single-organ studies, there is a need for careful control of PMIs for experimental and control samples in order to reduce the number of false discoveries. Due to a significant difference in dynamics and regulation of post-mortem proteome changes, it is important to avoid using samples from cadavers in studies involving cross-organ comparisons. Also, since PMI affects compartmentalization in a non-linear fashion, studies involving it should include only samples from living individuals. Our data also suggest that, in cadaver-based studies, the utility of antibody-based readouts may be limited, predominantly attributable to the impact of the protease activity, leading to the inability to detect epitopes despite the sustained presence of the protein.

## Material and methods

### In Vivo experiment and collection of the samples

All animal experiments were performed at the infection-free animal facility of the Uppsala University and approved by the Ethics Committee on Animal Experiments, Sweden (ethical approvals number 5.8.18-054012020). We have complied with all relevant ethical regulations for animal testing and research. Mice had free access to food and water (*ad libitum*). For the environmental enrichment, they were provided with hiding places and nesting material in each cage. The experimental protocol was designed prior the experiment and was not registered. For the experiment, mice (healthy, previously untreated, *Mus musculus*, NMRI, females, 6 months-old) were sedated with 4% Isofluran and, thereafter, euthanized by cervical dislocation. The heart, lung, spleen, liver, and kidney from each mouse were dissected (collected) at four different time points/conditions;

1. Immediately after termination
2. After keeping the bodies at room temperature for 5 h
3. After keeping the bodies at room temperature for 5 h and additional 24 h at +4 °C
4. After keeping the bodies at room temperature for 5 h and additional 4 days at +4 °C

The mouse bodies were kept in closed air-tight plastic bags with no light exposure and at the temperature mentioned above. No extra parameters were controlled in the process.

All organs except livers were placed in 1.5 ml Eppendorf tubes containing 1 ml All protect medium (AP, Qiagen). The livers were placed in 5 ml Eppendorf tubes containing 2 ml AP. After that, the tubes were stored according to the manufacturer’s instructions for AP.

### Homogenization of the organs

The organs were thawed on ice and washed 3 times in 10 ml PBS. Before homogenization, the organs were weighed and placed in microcentrifuge tubes (Qiagen, USA) containing 1 ml PBS with 0.1% Triton-x, with the exception of the liver that was placed in 100 µl of PBS with 1% Triton-x. 6× 3 mm tungsten beads (Qiagen, USA) were added to each tube, loaded in a Tissue Lyser LT (Qiagen, USA), and run at 50 rps for 20 min.

### SDC preparation of the samples

10% SDC solution was added to 30 µg of sample to make a final concentration of 1% SDC. The sample was then boiled at 95 °C for 10 min and sonicated for 2–4 s using a UW 2070(Bandelin) at 40% strength. The disulfide bonds were reduced by incubation at 37 °C for 30 min in the presence of 20 mM DTT (cas 3483-12-3, Sigma) and to alkylate the cysteines 40 mM Iodacetamide (Sigma) was added and incubated in darkness for 1 h. Trypsin was added, 1:100 w/w, and incubated at 37 °C overnight. The next day the samples were acidified using 1% formic acid and vortexed vigorously before being centrifuged at 16,500 × *g* for 20 min. The supernatant was further cleaned with C-18 tips as described below.

### Compartmentalization of the heart samples and digestion of the fractions

A part from each homogenized heart was compartmentalized using a Compartment Protein Extraction Kit (Merck, Germany) according to the manufacturer’s instructions; and cytosolic and ECM fractions were digested using an S-Trap column (Protifi, USA)^64^. To do that, 30 µg of a sample (measured using a BCA protein assay kit, see below) was mixed with a 20% SDS solution to a final concentration of 5% SDS. The sample was boiled at 95 °C for 10 min and then sonicated for 3 × 2 s using a UW 2070 (Bandelin) at 40% strength. This was followed by incubation at 37°C for 30 min in the presence of 20 mM DTT (Merck) and subsequently 40 mM Iodacetamide (Sigma) in darkness for 30 min. Phosphoric acid was added to the sample to a final concentration of 1%.

The sample was mixed with 7× the sample volume of 90% MeOH in 100 mM TEAB. The S-Trap column was loaded with 150 µl sample and spun at 4000 g for 30 s, this was repeated until all sample was loaded. The S-trap was washed 4 times with 150 µl 90% MeOH in 100 mM TEAB and transferred to a new collection tube. Trypsin was reconstituted in 50 mM TEAB and added to the column in a ratio of 1:50 (protein amount) and incubated O/N at room temperature. The peptides were eluted from the S-trap in 3 steps, first by addition of 40 µl 50 mM TEAB and spun at 1000 g 30 s, then 40 µl 0.2% formic acid (FA) was added and spun at 4000 g 30 s and last 40 µl of 50% acetonitrile (CAN) in 0.2% FA was added and at 4000 g 30 s. The ACN was removed from the sample using a SpeedVac (Savant, Thermo Fisher Scientific) and then washed with a C-18 column as described below.

### Cleaning of peptides with C-18

The peptides prepared by either S-trap or SDC preparation were subsequently cleaned using C-18 (10 µg capacity, Thermo Fisher Scientific) pipette tips according to the manufacturer’s instructions. In short, the C-18 tip was initialized by 80% ACN/ 5% FA and then re-equilibrated with 5% FA. The sample was loaded onto the C-18 tip after the addition of 5% FA, the C-18 tip was then washed once with 5% FA. The C-18 tip was transferred to a clean collection tube before the addition of 30% ACN in 5% FA and then 80% ACN in 5% FA. The eluted sample was dried using a SpeedVac and saved at −80 °C until further use.

### Bicinchoninic acid assay (BCA) assay to measure protein concentrations

The BCA protein kit (Pierce) was used according to the manufacturer’s instructions. In short, 10 µl of the samples or standard were pipetted to each well. The buffer A and B were mixed according to the manufacturer’s instructions no more than 10 min before use. The standard was mixed according to the company’s webpage (25, 125, 250, 500, 750, 1000, 1500, and 2000 µg/ml) and saved in −20 °C, then re-thawed and vortexed before use.

### Mass spectrometry analysis

The samples were analyzed using a Q Exactive Plus Orbitrap mass spectrometer (Thermo Fisher Scientific, Bremen, Germany) equipped with a nano-electrospray ion source. The peptides were separated via reversed-phase LC using an EASY-nLC 1000 system (Thermo Fisher Scientific). A set-up of a pre-column and an analytical column was used. The pre-column was a 2-cm EASY-Column (ID 100 µm, 5 µm C18; Thermo Fisher Scientific) and the analytical column was a 10-cm EASY-Column (ID 75 µm, 3 µm, C18; Thermo Fisher Scientific). Peptides were eluted with a 90 min linear gradient from 4% to 100% ACN at 250 nL min^−1^. During the gradient, all mass spectra were acquired in profile mode using the Orbitrap mass analyzer. An acquisition cycle consisted of one survey mass spectrum acquired from m/z 380 to 1120 Da, with a mass resolution of 70,000, an AGC target of 3,000,000, and a maximal injection time of 200 ms. This was followed by 34 DIA MS/MS events with 23 Da windows. The individual mass spectra were recorded at a resolution of 17,500 with 27% NCE and a maximal injection time of 50 ms.

### Bioinformatics and proteomic data analysis

Acquired raw files were converted to mzML format by MSConvert^65^ applying peak picking in the mass spectra with the vendor-provided algorithm (Thermo Fisher Scientific). The database search was performed in FragPipe (v20.0) using the mouse Swissprot database^66^. Trypsin with up to one missed cleavage was set as a digestion enzyme, and oxidation of methionine and acetylation of the N-terminus were set as variable modifications. Carbamidomethylation of cysteine residues was set as a fixed modification. Peptide length was restricted to 7–50 amino acids, and molecular mass from 500 to 5000 Da. For semi-tryptic peptide search, the enzyme-specific was defined as semi-specific with all other parameters kept constant. The resulting peptide-spectrum matches were adjusted to a 1% false discovery rate with Percolator^67^ as part of the Philosopher toolkit (v4.4)^68^ and converted to an MS/MS spectra library. DIA files were analyzed by DIA-NN 1.8.2 beta 27^69^.

### Statistics and reproducibility

All four experimental groups contained five animals (20 mice in total). The sample size was decided according to the current practice for mass spectrometry-based studies. No animals or data points were excluded from the analysis. No exclusion criteria were set a priori. The mass spectrometry sample preparations and the analysis were done without prior knowledge to the allocation of the experimental units. For the mass spectrometry sample preparations, the samples were randomized. All further data processing was performed in R. Known contaminants and decoy proteins were excluded from further analysis. Only proteins quantified in three out of the five replicates in at least one sample group were considered. Protein abundances were normalized by the median normalization approach and missing values at random were imputed by the Random Forest method, whereas missing values not at random were imputed by drawing values from a downshifted normal distribution^70^. This value was set to 3 valid quantifications per sample group, and its validity was confirmed through empirical exploration of the data. All statistical comparisons were performed based on a two-tailed Student’s *t*-test with equal variances; the differences between the comparisons were reported as log2-scaled fold changes. Proteins with a *p* value < 0.05 were considered significant. A bootstrapping approach was used to evaluate the false-positive rate, revealing a false discovery rate of less than 1%. Gene ontology analysis was performed with Metsacape using all quantified proteins as background^71^.

### Reanalysis of public proteomics data

Normalized and pre-processed proteomics data for Aryal et al. were downloaded from Massive (MSV000090320) the original publication and processed with limma with a linear mixed mode (assessing protein abundance, patient diagnosis, sex, rate of death, and the PMI) as described in the original publication^42^. For Ping et al. results from files from Proteome Discoverer were downloaded from Pride (PXD020296)^43^. Proteins were filtered for “Master proteins” and proteins with at least 2 peptides, abundances were corrected by median normalization. Differential protein analysis was performed as in Aryal et al. (assessing protein abundance, patient diagnosis, sex, and the PMI). For the assessment of semi-tryptic protein changes in human post-mortem samples, data from Kocsmár et al. were downloaded and searched with MSFragger with the same settings as the in-house mouse data^41^.

## Supplementary information

Supplementary Information

Supplementary Data 1

Supplementary data 2

Supplementary data 3

Supplementary data 4

Supplementary data 5

Supplementary data 6

Supplementary Data 7

Description of Additional Supplementary Files

## Data Availability

The mass spectrometry proteomics data files have been deposited to ProteomeXchange Consortium (http://proteomecentral.proteomexchange.org) via the PRIDE partner repository^72^ with the data identifier PXD053048. Metadata relating to the individual raw files can be found on PRIDE or in Supplementary Data 6. The source data behind the graphs and charts in the paper can be found in Supplementary Data 7. All other data are available from the corresponding author on reasonable request.
