# Supplementary material for: A multi-tissue longitudinal proteomics study to evaluate the suitability of post-mortem samples for pathophysiological research
Source: Commun Biol. 2025 Jan 17;8:78. doi: 10.1038/s42003-025-07515-z (PMC11742016; doi:10.1038/s42003-025-07515-z)
Supplement: Supplementary file 1 — Supplementary Information [file 42003_2025_7515_MOESM1_ESM.pdf]

Supplementary Information

Supplementary Figure 1

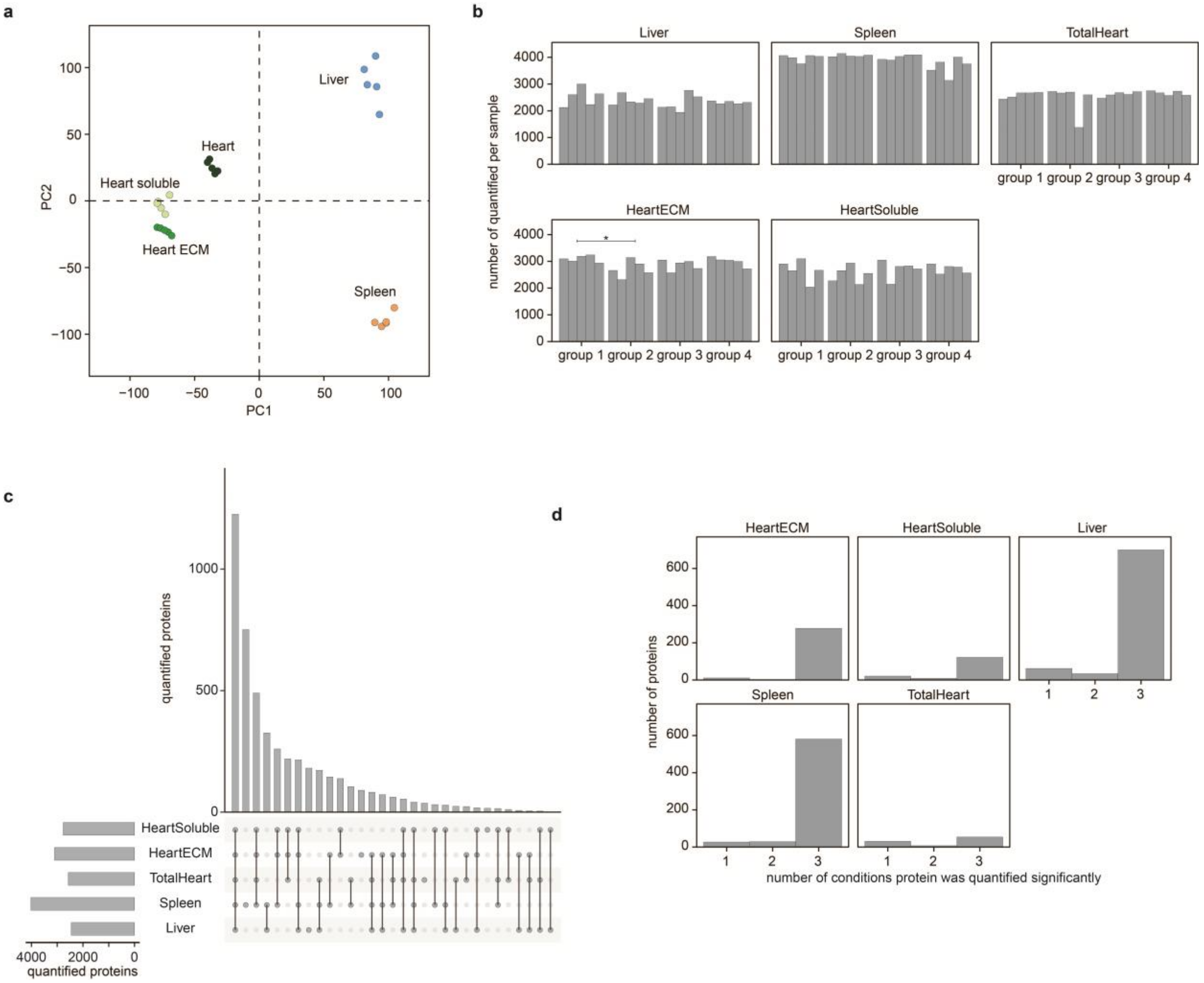

Supplementary Figure 1: Assessment of proteomics data quality. a) Principal component analysis (PCA) across all fresh proteomics samples. **b)** Number of quantified proteins in the different fresh tissue samples. **c)** Upset plot assessing the protein identification across the different tissues. **d)** The number of occurrences of a protein was significantly changing abundance compared to fresh samples based on ANOVA analysis. N = 5 for all mouse proteomics samples.

Supplementary Figure 2

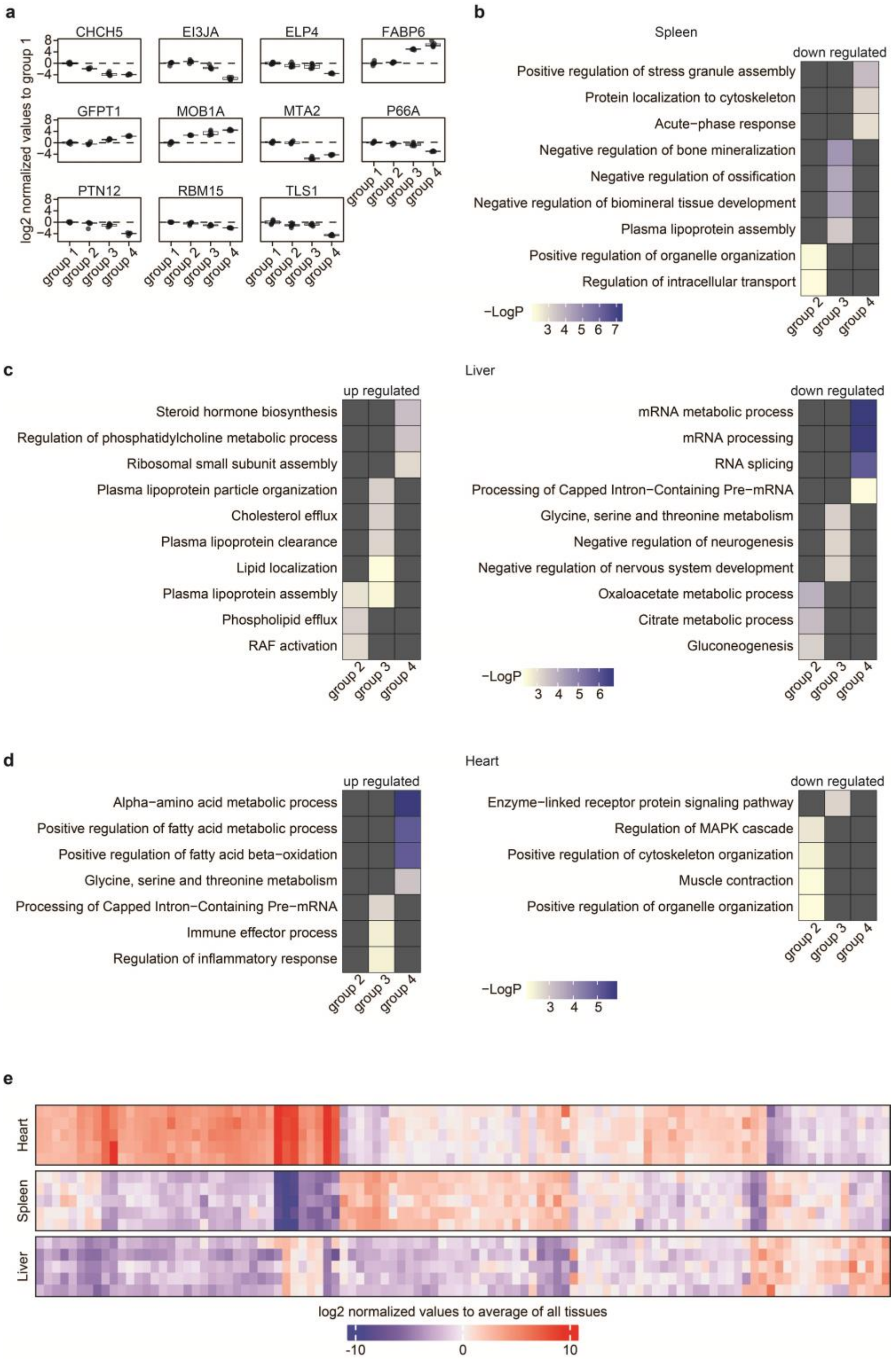

Supplementary Figure 2: Assessment of tissue-specific proteome changes post-mortem. a) Relative protein abundance changes of most significant changing protein in the liver post-mortem. **b)** Gene ontology analysis of downregulated proteins in the spleen in group 4 compared to group 1 (fresh). Gene ontology analysis of differentially regulated proteins in the liver **c)** or heart **d)** in group 4 compared to group 1 (fresh). **e**) Expression of sarcomere proteins in fresh samples across the three organs. The horizontal

line in the boxplots represents the median, 25th, and 75th percentiles and the whiskers represent measurements to the 5th and 95th percentiles. N = 5 for all mouse proteomics samples.

Supplementary Figure 3

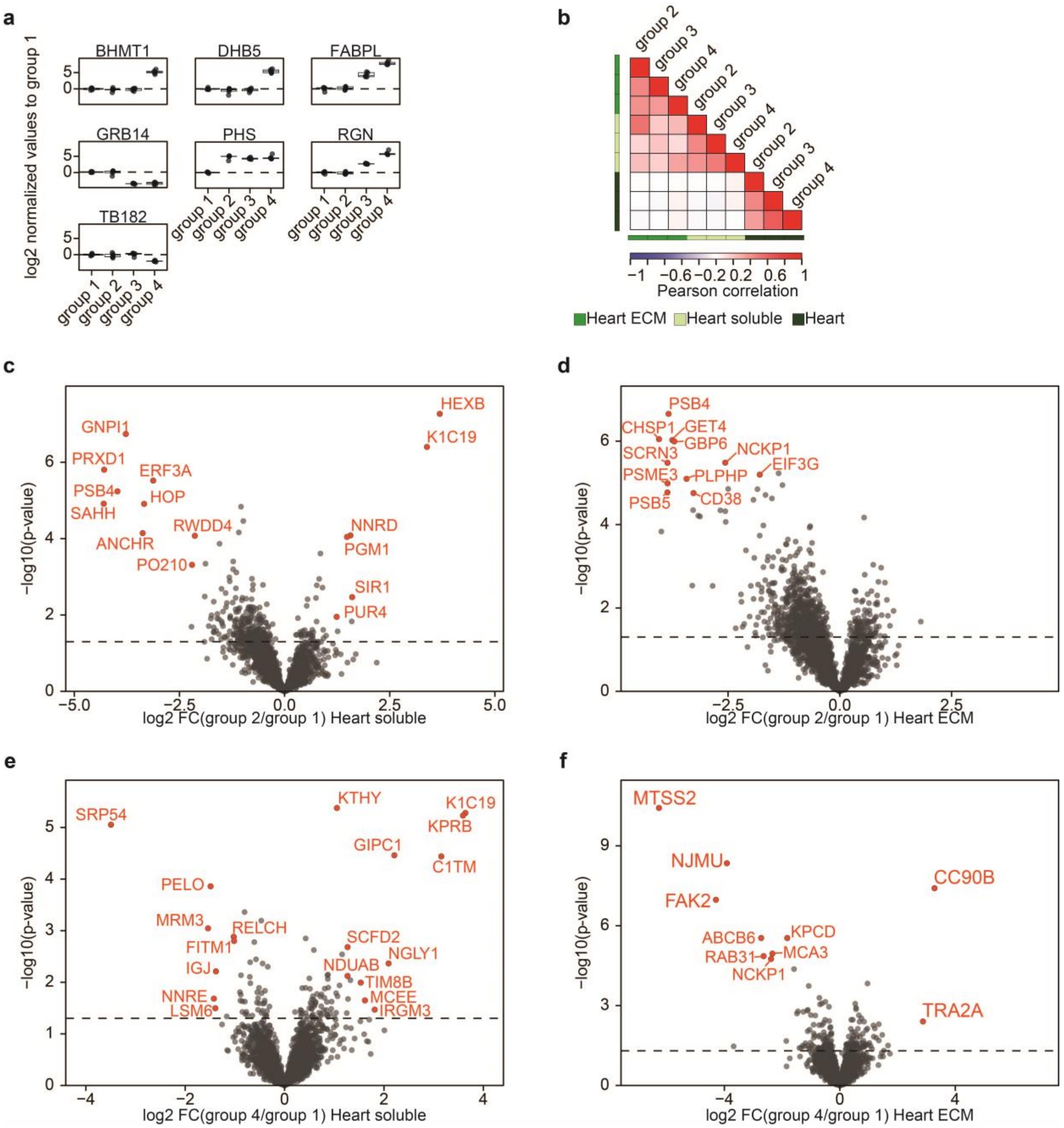

Supplementary Figure 3: Protein alterations in the total heart and different subcellular fractions. a) Boxplot of proteins significantly changing abundance in the heart in group 4 compared to fresh samples. **b)** Correlation between the different heart fractions and sampling points. Volcano plot highlighting differentially abundant protein in the soluble **c)** and ECM heart fraction **d)** between fresh samples (group 1) and samples incubated for 5h at room temperature (group 1). Volcano plot highlighting differentially abundant protein in the soluble **e)** and ECM heart fraction **f)** between fresh samples (group 1) and samples incubated for 4 days (group 4). P-values were calculated using a two-sided Student t-test using equal variance and a $p < 0.05$ was considered as significant. The horizontal line in the boxplots represents the median, 25th, and 75th percentiles and the whiskers represent measurements to the 5th and 95th percentiles. N = 5 for all mouse proteomics samples.

Supplementary Figure 4

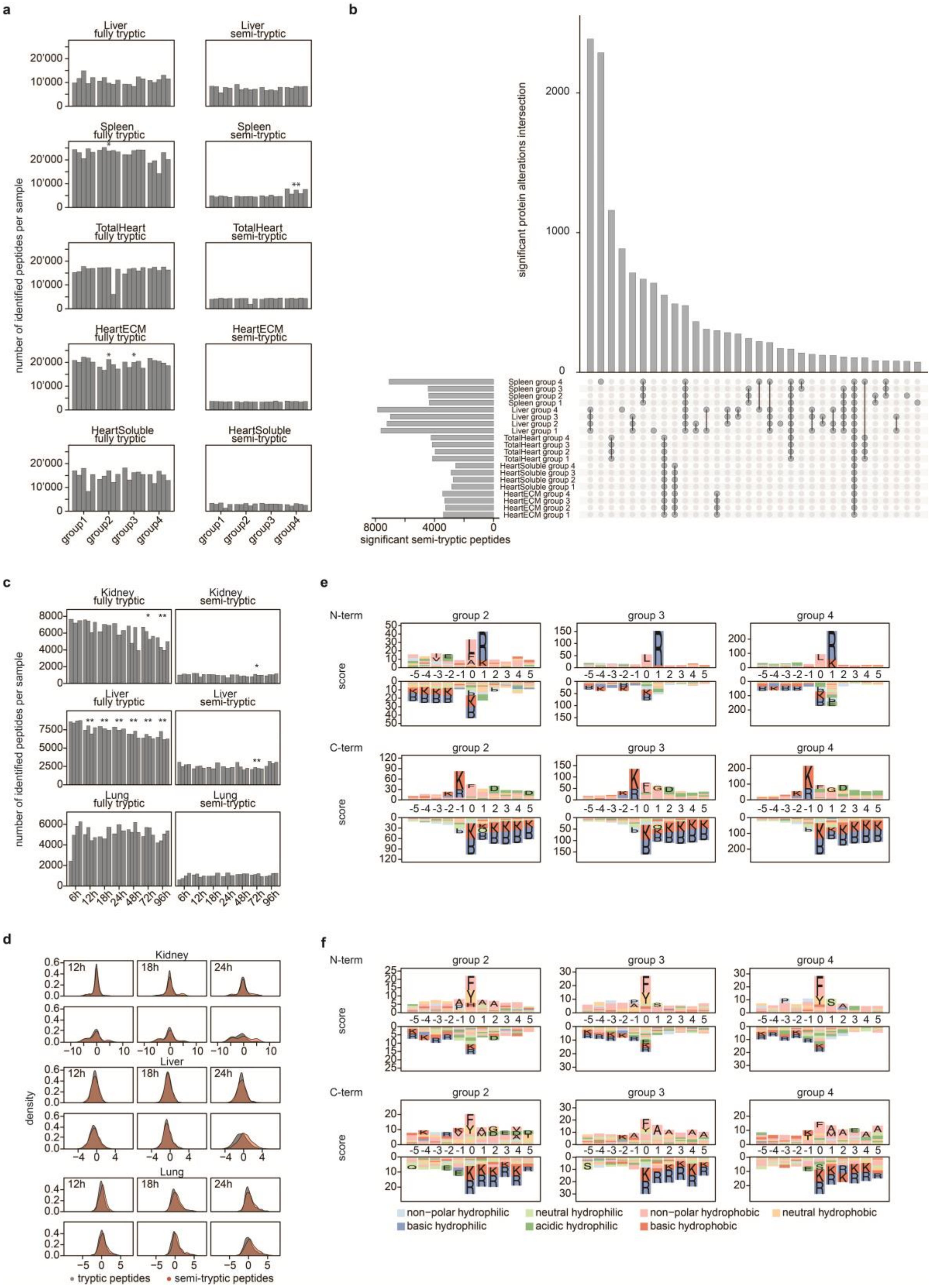

Supplementary Figure 4: Analysis of peptide integrity post-mortem. a) Number of peptides with different cleave specificity in different mouse tissues post-mortem**. b)** Overlap of semi-tryptic peptides across the different organs and sample groups. **c)** Number of tryptic and semi-tryptic peptides in human cadaver samples by Kocsmár et al. **d**) Distribution changes of tryptic and semi-tryptic peptides abundances in human cadaver samples by Kocsmár et al. Cleavage specificity for semi-tryptic peptides in the mouse liver **e)** and heart **f).** N = 5 for all mouse proteomics samples.

Supplementary Figure 5

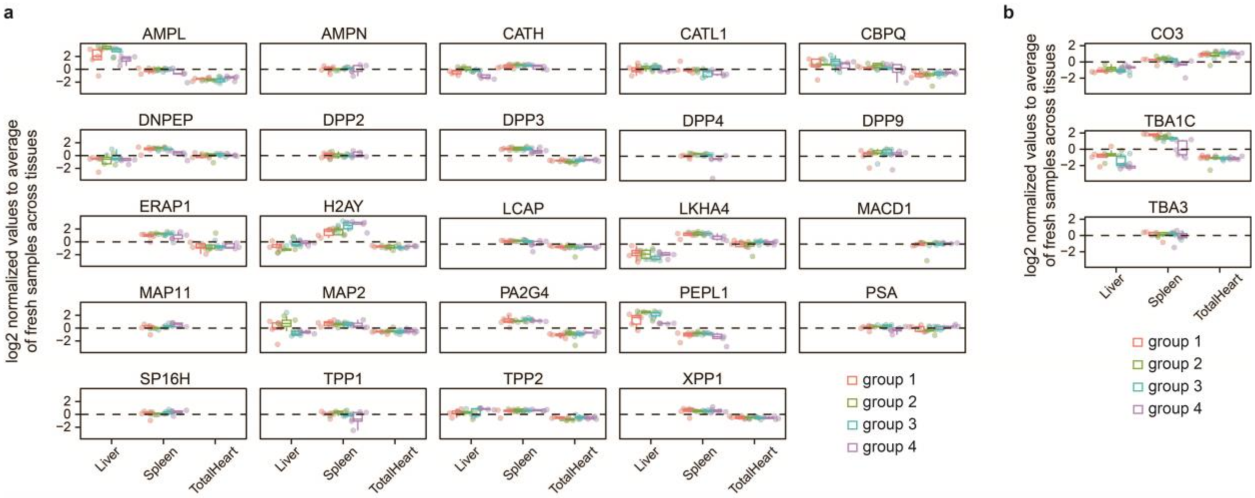

Supplementary Figure 5: Analysis of peptidases. Quantification of exopeptidases **a**) and endopeptidases **b**) in fresh tissue, comparing levels across different organs. The horizontal line in the boxplots represents the median, 25th, and 75th percentiles and the whiskers represent measurements to the 5th and 95th percentiles. N = 5 for all mouse proteomics samples.
