# Supplementary material for: A multi-tissue longitudinal proteomics study to evaluate the suitability of post-mortem samples for pathophysiological research
Source: Commun Biol. 2025 Jan 17;8:78. doi: 10.1038/s42003-025-07515-z (PMC11742016; doi:10.1038/s42003-025-07515-z)
Supplement: Supplementary file 9 — Description of Additional Supplementary Files [file 42003_2025_7515_MOESM9_ESM.pdf]

Description of Additional Supplementary Files

File name: Supplementary Data 1
Description: Results from reanalysis from Ping et al.

File name: Supplementary Data 2
Description: Results from reanalysis from Aryal et al.

File name: Supplementary Data 3
Description: ANOVA results from tissue time-course degradation analysis

File name: Supplementary Data 4
Description: Statistical analysis from all pairwise proteome abundance alterations

File name: Supplementary Data 5
Description: Statistical results from all semi-tryptic peptide analysis

File name: Supplementary Data 6
Description: Annotation list for proteomics raw files

File name: Supplementary Data 7
Description: Source data for graphs and charts
